# Decoupling Research of a Novel Three-Dimensional Force Flexible Tactile Sensor Based on an Improved BP Algorithm

**DOI:** 10.3390/mi9050236

**Published:** 2018-05-14

**Authors:** Yang Song, Feilu Wang, Zhenya Zhang

**Affiliations:** 1 School of Electronics and Information Engineering, Anhui Jianzhu University, Hefei 230601, China; esunny@ahjzu.edu.cn (Y.S.); zzychm@ustc.edu.cn (Z.Z.); 2 School of Electronics and Information Engineering, Anhui University, Hefei 230601, China; 3 Anhui Key Laboratory of Intelligent Building, Anhui Jianzhu University, Hefei 230022, China

**Keywords:** tactile sensor array, three-dimensional force, tactile sensing, deformation, decoupling method, BP algorithm

## Abstract

Decoupling research on flexible tactile sensors play a very important role in the intelligent robot skin and tactile-sensing fields. In this paper, an efficient machine learning method based on the improved back-propagation (BP) algorithm is proposed to decouple the mapping relationship between the resistances of force-sensitive conductive pillars and three-dimensional forces for the 6 × 6 novel flexible tactile sensor array. Tactile-sensing principles and numerical experiments are analyzed. The tactile sensor array model accomplishes the decomposition of the force components by its delicate structure, and avoids direct interference among the electrodes of the sensor array. The force components loaded on the tactile sensor are decoupled with a very high precision from the resistance signal by the improved BP algorithm. The decoupling results show that the *k*-cross validation (*k*-CV) algorithm is a highly effective method to improve the decoupling precision of force components for the novel tactile sensor. The large dataset with the *k*-CV method obtains a better decoupling accuracy of the force components than the small dataset. All of the decoupling results are fairly good, and they indicate that the improved BP model with a strong non-linear approaching ability has an efficient and valid performance in decoupling force components for the tactile sensor.

## 1. Introduction

With the rapid developments of intelligent robots and tactile-sensing technology, soft and thin tactile sensors play a very important role in the intelligent robot skin field, as they can offer feedback information about external forces loaded on the robot skin. Tactile sensors can help robots recognize objects and forces loaded on to complete a variety of complex tasks. They can acquire lots of useful information—such as shapes, hardness, elasticity, and roughness—from the object and the surrounding environment. In practice, flexible tactile sensors are supposed to be wearable and used as robot skin through which people can detect the tactile force loaded on the robot. The sensitivity of tactile sensing is crucial for ensuring the safe and efficient interaction between robots and the environment; tactile sensor research plays an indispensable role in the bionic intelligent robot field.

The design principle for tactile sensors developed with different tactile-sensing mechanisms mainly concentrate on piezoelectricity [1,2], capacitance [3,4,5,6], optical fiber [7,8,9], single-walled carbon nanotube [10] technology, and so forth. For most of the tactile sensors, their sensing surface is divided into scattered areas without a continuous elastomer skin, and the sensing units are usually independent of each other. Zhenan et al. [11] proposed skin-like pressure and strain sensors based on the transparent elastic films of carbon nanotubes, which can measure the deformation generated by a butterfly standing on the surface. Asadnia [12] developed a highly stretchable, self-powered, and ultra-sensitive strain sensor based on piezoelectric polyvinylidene fluoride (PVDF) nanofibers. The flexible strain sensor has a good performance in human motion recognition by fabricating a glove with two sensors mounted on the middle and index fingers. Harada et al. [13] illustrated a fully printed fingerprint-like three-axis tactile force and temperature sensor for artificial skin application. The high sensitivity and selectivity for both force and temperature are illustrated by using a 3 × 3 array artificial skin that can sense tactile, slip, friction, and temperature. Weiting et al. [14] utilized the diffusion effect of an elastomer cover to identify an arbitrary contact force based on a 3 × 3 sparse tactile sensor array, which is also feasible to realize density load detection. Cabibihan [15] investigated various thicknesses of artificial skin, and found that it blurs the mechanical signals transmitted to the embedded sensor. Additionally, some other studies have concentrated on the grasp control for specific environments by estimating the contact force [16,17], but paid less attention to the decoupling method for the force loaded on the sensor.

As the coupling relationship generally exists among the high-dimensional variables of the tactile sensor, more and more researchers have focused on the intelligent decoupling methods by using types of efficient machine learning methods to decouple the relationship among the high-dimensional information of the tactile sensor. Aiguo Song [18] proposed a static decoupling algorithm for a six-axis force sensor based on coupling errors and the piecewise fitting model. Lee et al. [19] proposed a new type of slim and flexible tactile sensor. A triaxial force decoupling algorithm was improved by combining two-dimensional mapping data calculated by finite element analysis, which is able to measure the triaxial forces using only four strain gauges. Wang et al. [20] decoupled the three-dimensional information of a special tactile sensor by a radial basis function machine-learning algorithm under ideal conditions. Chen et al. [21] put forward a kind of data decoupling algorithm for the overvoltage sensor based on interphase coupling theory, and gained high precision.

Based on the above analysis, this paper focuses on the study of the multi-dimensional information decoupling and the approximation of the high dimensional non-linear mapping relationship between the resistances signal and three-dimensional force components for a novel flexible tactile sensor array. In order to gain a high precision for the decoupling result, the improved back-propagation (BP) models with different hidden nodes based on the *k*-cross validation (*k*-CV) method are constructed in different ways to decouple the force components for the tactile sensor. This paper is organized as follows. Section 2 analyzes the detection principles, constructs the mathematical model for the novel flexible tactile sensor, and presents the improved BP decoupling method for the force components. Section 3 elaborates the decoupling process and discusses the decoupling results for the force components applied to the tactile sensor based on an improved BP algorithm with different strategies. The decoupling experiments for the tactile sensor array with different methods are carried out in Section 3. Finally, Section 4 presents the conclusions of this paper.

## 2. Principles and Methods

### 2.1. Properties of Conductive Rubber

Conductive rubber is used as the main material of the flexible tactile sensor. It is made by conductive particles (such as carbon black) that are uniformly mixed and dispersed in silicone rubber material. It has pretty good features, such as flexibility, force-sensitivity, conductivity, and low cost. The conductive rubber has a very good force-sensitive property, since its conductivity is changed with the change of force or pressure. If there is no force applied, the gaps among the conductive particles are large, and the conductive rubber has high resistivity and low conductivity. However, with the force loaded on the conductive rubber, the gaps among the conductive particles become smaller, which lead to lower resistivity and higher conductivity. This characteristic of the conductive rubber is the force-sensitive property. Therefore, the conductive rubber is called the force-sensitive conductive rubber. It is an important and efficient material in the flexible tactile sensor manufacture field.

### 2.2. Prototype of the Tactile Sensor Array

In this paper, the main material of the flexible tactile sensor is the force-sensitive conductive rubber, which is a kind of conductive polymer. The conductive rubber was made by the CB3100 carbon black uniformly dispersed in the silicone rubber. Firstly, the CB3100 carbon black material configured with the volume fraction of 28% is placed in the model, and the petroleum ether is added. Then, the carbon black filler is dispersed using an ultrasonic disperser for 20 min. After that, the silicone rubber matrix is added and uniformly dispersed together with the carbon black for 30 min. Furthermore, it is placed in a thermostatic chamber at a room temperature of 25 °C for 48 h. Finally, after demolding, the conductive rubber samples are obtained. The parameters of the conductive rubber are shown in Table 1.

The flexible tactile sensor is manufactured mainly by using the conductive rubber as the conductive component and using the silicon rubber as the matrix. It is a force-sensitive, conductive, flexible, and wearable tactile sensor. It is suitable to be used as the robot skin. In this paper, the force-sensitive conductive rubber is regarded as an elastomer with continuous, isotropic, repetitive, and no hysteresis properties.

**Figure 1:**
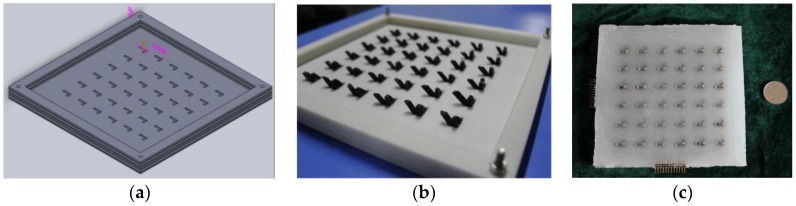
(**a**) The model drawn by the Solidworks software; (**b**) the three-dimensional (3D) printed mold; (**c**) the prototype of the tactile sensor.

**Figure 2:**
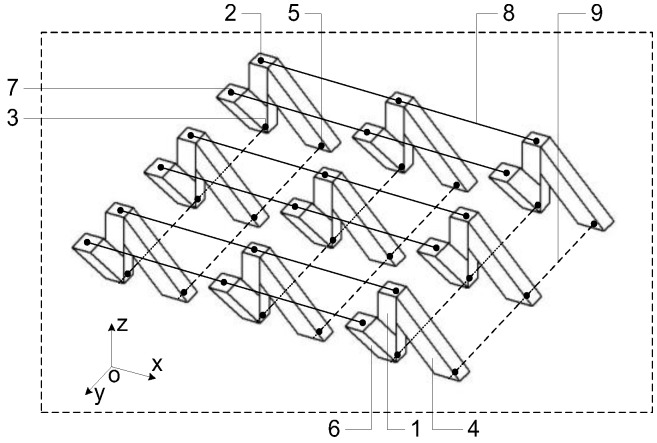
The flexible tactile sensor array model.

In order to solve the coupling problems among multi-dimensional tactile information, and improve the information detection ability and decoupling precision for the tactile sensor, this paper presents a novel three-dimensional force flexible tactile sensor with a particular structure based on the force-sensitive conductive rubber material. Firstly, the Solidworks (SolidWorks 2014, SolidWorks Inc., Waltham, MA, USA) is utilized to draw the model for the sensor array, as shown in Figure 1a. Secondly, the three-dimensional (3D) printing technology is used to produce that model for a 6 × 6 tactile sensor array, which is shown in Figure 1b. Thirdly, the novel tactile force-sensitive units made by the conductive rubber are put into the meshes of the model, as shown in Figure 1b, and the spaces between the sensitive units are filled with the insulating silicon rubber material. Then, the flexible wires that connect the sensitive units made by the conductive rubber are arranged and encapsulated in the model by the insulating silicon rubber. After that, the model is put into the thermostatic chamber at the temperature of 25 °C for 48 h. The wires are shown in Figure 2, and they are represented by “8” and “9”. Finally, after demolding, the prototype of the novel tactile sensor fabricated with conductive rubber and silicon rubber materials is obtained, as shown in Figure 1c.

The 3D printing technology reduces the instability and difficulty during the fabrication process, and improves the consistency and accuracy of each tactile force-sensitive microstructure unit. It can realize the standardized manufacture for 3D flexible tactile sensor arrays. With the novel structure, the three-dimensional tactile force applied on the sensor can be decomposed directly.

### 2.3. Structure of the Flexible Tactile Sensor

When an external force is applied on the novel tactile sensor, the three-dimensional tactile force would be transmitted into the three conductive pillars of the sensitive unit and can be decomposed into three components—*Fx*, *Fy*, and *Fz*—directly. The complexity and non-linearity of the sensor model are reduced by its particular structure and manufacture process. Accordingly, the high-dimensional information of the sensor could be decoupled quickly and accurately with high precision and low relative error.

The model of the novel tactile sensor array illustrated in Figure 1b is shown in Figure 2. It is composed of three parts. The first part is the tactile-sensing array, which consists of microstructure sensitive units made by soft conductive rubber, as shown in Figure 2. The second part is the framework made by the flexible insulating silicon rubber material. The spaces between the sensitive units are filled with the silicon rubber. The third part is electrical wires that connect the microstructure sensitive units and the external acquisition circuit.

**Figure 3:**
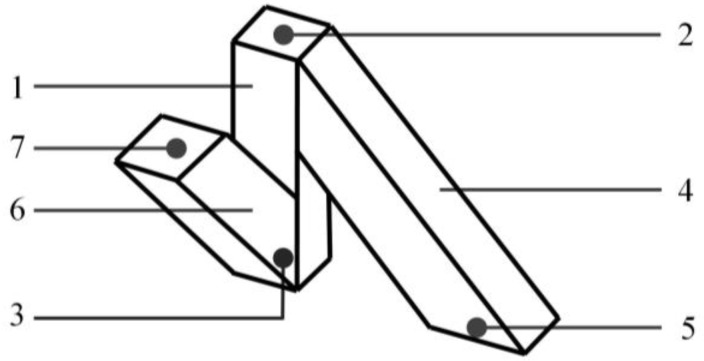
The microstructure sensitive unit.

The key component of the tactile sensor array is the orderly arranged flexible microstructure tactile force-sensitive unit that is shown in Figure 3, which acts as the transmission medium of the three-dimensional force. The sensitive unit is the main force-sensitive component of the sensor. It is composed of three flexible conductive pillars made by the tactile force-sensitive conductive rubber.

In Figure 2 and Figure 3, “1”, “4”, and “6” denote the three conductive pillars made by force-sensitive conductive rubber. The “2” and “7” denote the tactile-sensing electrodes on the upper layer, and “3” and “5” denote the tactile-sensing electrodes on the lower layer. Spaces between the different microstructure sensitive units are filled up by the insulating silicon rubber. Originally, the length of pillar “1”, pillar “4”, and pillar “6” were 1 cm, $\sqrt{2}$ cm, and $\sqrt{2}$ cm, respectively. The angle between pillar “1” and pillar “4” and between pillar “1” and pillar “6” are both 45°. The cross-sectional area of each pillar is 4 mm^2^. The “8” represents the row wires connecting the upper layer sensing electrodes by rows. The “9” represents the column wires connecting the lower layer sensing electrodes by columns. The upper row wires and the lower column wires are perpendicular to each other.

### 2.4. Detection Principle and Mathematical Model

As the force is loaded on the sensor, the insulating rubber near the stress area would be extruded or stretched. Meanwhile, the force would be transferred into sensitive units, which are close to the stress area. Accordingly, the length and the resistance of the corresponding conductive pillars would be changed. The resistances can be detected by sequentially scanning the resistance between each row wire, and all the column wires. Then, the three-dimensional force would be decoupled by the changed resistances based on machine learning algorithms.

**Figure 4:**
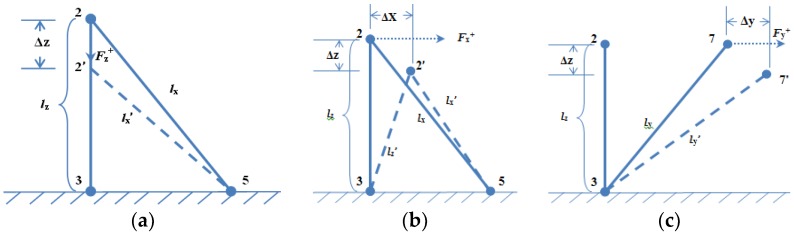
(**a**) The displacement of the micro sensitive unit along *Z* direction; (**b**) the displacement of the micro sensitive unit along *X* direction; (**c**) the displacement of the micro sensitive unit along *Y* direction.

It is assumed that the lower layer of the sensor has no deformation, and the lower electrodes have no displacement. When the external force is applied on the sensor, the position of the sensing electrodes “2” or “7” in the upper layer would be changed. The schematic diagram of the displacement of the sensitive unit is shown in Figure 4. We can firstly deduce and solve the length variation for each pillar (Δ*l_i_*, *i* = *x*, *y*, *z*) through the changing resistances. Then, we can emulate the deformation or displacement of the upper electrodes. At last, we can approach the mathematical relationship between the resistances and three-dimensional force and decouple the force components loaded on the tactile sensor by the intelligent BP algorithm, which is one of the most popular and classical machine learning methods.

The conductive rubber that we studied is shown as an elastomer. It is supposed that the volume of each conductive pillar has not changed when the force is loaded. Accordingly, the resistances of the three conductive pillars of each sensitive unit can be calculated by the equations as follows:

(1) $$R_{i}=\rho \frac{l_{i}}{S}=\rho \frac{l_{i}^{2}}{V_{i}},i=x,y,z$$

where, *Rz*, *Rx*, and *Ry* denote the original resistance of the pillar labeled “1”, “4”, and “6”, respectively.

When a three-dimensional force is applied, the changed length of the conductive pillars can be calculated by detecting the corresponding resistance as below:

(2) $$l_{i}^{\prime }=l_{i}\sqrt{R_{i}^{\prime }/R_{i}},i=x,y,z$$

Whence:

(3) $$\begin{array}{l} \Delta l_{z}=|l_{z}-l_{z}^{\prime }|=\sqrt{V_{z}/\rho }|\sqrt{R_{z}^{\prime }}-\sqrt{R_{z}}|\\ \Delta l_{x}=|l_{x}-l_{x}^{\prime }|=\sqrt{V_{x}/\rho }|\sqrt{R_{x}^{\prime }}-\sqrt{R_{x}}|\\ \Delta l_{y}=|l_{y}-l_{y}^{\prime }|=\sqrt{V_{y}/\rho }|\sqrt{R_{y}^{\prime }}-\sqrt{R_{y}}| \end{array}$$

where, ∆*l_x_*, ∆*l_y_*, and ∆*l_z_* denote the deformation of conductive pillars labeled “4”, “6”, “1”, respectively.

The deformation of the sensitive unit could be gained through the particular analysis. For each microstructure tactile force-sensitive unit, it has three output components: $R_{x}^{\prime }$, $R_{y}^{\prime }$, and $R_{z}^{\prime }$. The input of the sensitive unit is the force that can be decomposed into three components: *Fx*, *Fy*, and *Fz*. As the conductive rubber has a piezoresistive effect, and it satisfies the condition that *F* ∝ ∆*l*, the relationship between the three-dimensional force and resistances can be obtained approximately as follows:

(4) $$\begin{array}{l} F_{z}\propto \sqrt{V_{z}/\rho }|\sqrt{R_{z}^{\prime }}-\sqrt{R_{z}}|\\ F_{x}\propto \sqrt{V_{x}/\rho }|\sqrt{R_{x}^{\prime }}-\sqrt{R_{x}}|\\ F_{y}\propto \sqrt{V_{y}/\rho }|\sqrt{R_{y}^{\prime }}-\sqrt{R_{y}}| \end{array}$$

It is also assumed that the conductive pillar satisfies the generalized Hooke’s law. Therefore, the relationship between stress and strain can be defined as:

(5) $$\sigma =E\frac{\Delta l}{l}$$

Additionally:

(6) $$E\frac{\Delta l}{l}=\frac{F}{S}$$

where $\Delta l=|l^{\prime }-l|$, $l^{\prime }$ denotes the pillar’s length when it is suffered by stress.

(7) $$F=\frac{E\cdot S}{l}\Delta l$$

The substitution of Equation (3) into Equation (7) gives an expression for the components of stress at any point, these are the following:

(8) $$F_{i}=\frac{E\cdot S}{l_{i}}\sqrt{V_{i}/\rho }|\sqrt{R_{i}^{\prime }}-\sqrt{R_{i}}|,i=x,y,z$$

So, the relationship between the three-dimensional force and resistance of each conductive pillar can be calculated by Equation (8). It can be generalized as:

(9) $$F_{i}=f_{m}(R_{i}^{\prime })=g_{m}(\Delta l_{i}),i=x,y,z;m=1,2,3$$

So far, the flexible tactile sensor with its delicate structure accomplishes the decomposition of three-dimensional information. It avoids the direct interference among the sensing electrodes on the conductive pillars, and reduces the complexity and non-linearity of the tactile sensor. Meanwhile, the high-dimensional information of the sensor would be decoupled with high precision based on an improved BP algorithm. As the length of the conductive pillar is very short, and its deformation is small, which is not more than 30% of the pillar’s length, it can be approximately considered as ∆*l_i_* ≅ ∆*i*, *i* = *x*, *y*, *z*, where ∆*i* is the displacement/deformation of the upper sensing electrodes along the *i* direction.

As the resistance of conductive pillars can be acquired from the external circuit, this paper takes full advantage of the intelligent BP algorithm and its strong function approximation ability to approach the mapping relationship between the resistances and three-dimensional force. Then, the tactile force can be well decoupled by the resistance of conductive pillars.

### 2.5. Analytical Analysis

The displacements of the sensing electrodes on the upper surface can be obtained from the sensor array. The status of the tactile force can be gained from the information of several discrete sensing electrodes. With regard to general elastomers, a potential theory [22] had resolved how to get the deformation of the whole elastomer through the distribution of the force loaded on the elastomer, which is supposed as a half-space elastomer. That conclusion is also suitable for the kind of flexible tactile sensor that is studied in this paper.

**Figure 5:**
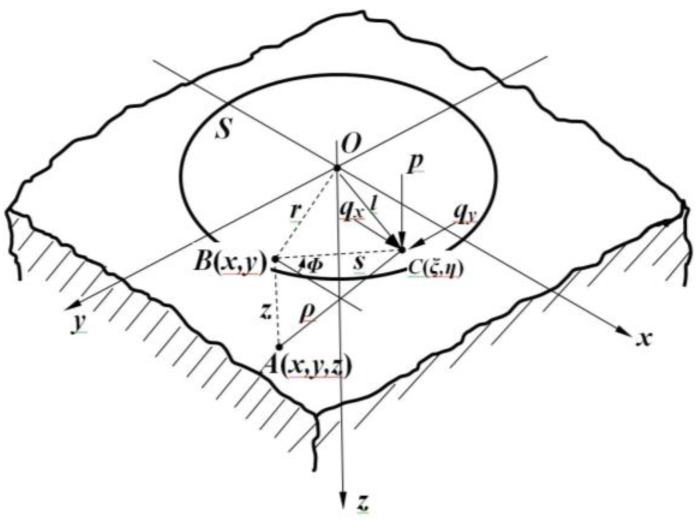
The schematic diagram of half-space when force is loaded.

The half-space is shown in Figure 5. *C*(*ξ*, *η*) is taken as a general surface point within the loaded area *S*, whilst *A*(*x*, *y*, *z*) is a general point within the body of the elastomer; then, the distance between *A* and *C* is defined as *CA* ≡ *ρ* = {(*ξ* − *x*)^2^ + (*η* − *y*)^2^ + *z*^2^ }^1/2^.

The distributions of one normal component and two shear components of the three-dimensional force acting on the area *S* are supposed as *p*(*ξ*, *η*), *q_x_*(*ξ*, *η*), and *q_y_*(*ξ*, *η*), and the potential functions that are satisfied to Laplace’s equations are defined as follows:

(10) $$\begin{array}{l} F_{1}=\overset{\;}{\underset{S}{\int }}\overset{\;}{\underset{\;}{\int }}q_{x}(\xi ,\eta )\Omega d\xi d\eta \\ G_{1}=\overset{\;}{\underset{S}{\int }}\overset{\;}{\underset{\;}{\int }}q_{y}(\xi ,\eta )\Omega d\xi d\eta \\ H_{1}=\overset{\;}{\underset{S}{\int }}\overset{\;}{\underset{\;}{\int }}p(\xi ,\eta )\Omega d\xi d\eta \end{array}$$

where Ω = *z*ln(*ρ* + *z*) − *ρ*. In addition, the potential functions are defined as follows:

(11) $$\begin{array}{l} F=\frac{\partial F_{1}}{\partial z}=\overset{\;}{\underset{S}{\int }}\overset{\;}{\underset{\;}{\int }}q_{x}(\xi ,\eta )\ln(\rho +z)d\xi d\eta \\ G=\frac{\partial G_{1}}{\partial z}=\overset{\;}{\underset{S}{\int }}\overset{\;}{\underset{\;}{\int }}q_{y}(\xi ,\eta )\ln(\rho +z)d\xi d\eta \\ H=\frac{\partial H_{1}}{\partial z}=\overset{\;}{\underset{S}{\int }}\overset{\;}{\underset{\;}{\int }}p(\xi ,\eta )\ln(\rho +z)d\xi d\eta \end{array}$$

Let φ1=∂F1∂x+∂G1∂y+∂H1∂z, φ=∂φ1∂z=∂F ∂x+∂G ∂y+∂H ∂z.

The potential theory shows that when the force is loaded, the components of the elastic displacement or deformation *u_x_*, *u_y_*, and *u_z_* at any point *A*(*x*, *y*, *z*) inside of the elastomer can be expressed in terms of the above functions as follows:

(12) $$\begin{array}{l} u_{x}=\frac{1}{4\pi G}(2\frac{\partial F}{\partial z}-\frac{\partial H}{\partial x}+2\nu \frac{\partial \varphi_{1}}{\partial x}-z\frac{\partial \varphi }{\partial x})\\ u_{y}=\frac{1}{4\pi G}(2\frac{\partial G}{\partial z}-\frac{\partial H}{\partial y}+2\nu \frac{\partial \varphi_{1}}{\partial y}-z\frac{\partial \varphi }{\partial y})\\ u_{z}=\frac{1}{4\pi G}(\frac{\partial H}{\partial z}-(1-2\nu )\varphi -z\frac{\partial \varphi }{\partial z}) \end{array}$$

These expressions decrease as 1/*ρ* at large distances from the loaded region. Therefore, the elastic deformation/displacements of the points are close to the loaded region relative to the points in the elastomer at a large distance from the loaded region (ρ→ ∞) where the half-space can be looked upon as fixed.

The displacements having been found, the stresses can be calculated from the corresponding strains by Hooke’s law. Under the action of a purely normal pressure *p*(*ξ*, *η*), which would occur in a frictionless contact, the above equations can be simplified as follows:

(13) $$\begin{array}{l} u_{x}=-\frac{1}{4\pi G}((1-2\nu )\frac{\partial \varphi_{1}}{\partial x}+z\frac{\partial \varphi }{\partial x})\\ u_{y}=-\frac{1}{4\pi G}((1-2\nu )\frac{\partial \varphi_{1}}{\partial y}+z\frac{\partial \varphi }{\partial y})\\ u_{z}=\frac{1}{4\pi G}(2(1-\nu )\varphi -z\frac{\partial \varphi }{\partial z}) \end{array}$$

where, $\varphi_{1}=\frac{\partial H_{1}}{\partial z}=\overset{\;}{\underset{S}{\int }}\overset{\;}{\underset{\;}{\int }}p(\xi ,\eta )\ln(\rho +z)d\xi d\eta ,\varphi =\overset{\;}{\underset{S}{\int }}\overset{\;}{\underset{\;}{\int }}p(\xi ,\eta )\frac{1}{\rho }d\xi d\eta$.

The above equations provide a formal solution to the problem of stress and deformation in an elastic half-space with prescribed tractions acting on the surface. If the distribution of tractions within the area *S* is known explicitly, then, in principle, the stress and displacement/deformation at any point in the elastomer can be found. In practice, obtaining an expression in closed form for the stresses in any but the simplest problems present difficulties. In particular circumstances, more sophisticated analytical techniques have been developed to overcome some of the difficulties of the classical approach.

In view of that, the stress and displacement generated by the concentrated normal and tangential forces would be conducted to simplify the problem. The stress distribution and deformation resulting from any distributed loading can then be found by the superposition principle.

According to the superposition principle [23], the mathematical relationship between the force components and deformation or displacement of the elastomer can be linearly superimposed. If displacement D1 and D2 are generated by the force component F1 and F2 separately, accordingly, the displacement D1 + D2 can be generated by F1 + F2.

The stresses and displacements generated by a concentrated point force *P* acting normally to the surface at the origin can be found directly in several ways. It is assumed that the three-dimensional force loading region is around the original point, and that the area of the loading region is very small, which makes *ρ*, in the above equations, become a constant that does not include variables *ξ* and *η*. Then, Equation (13) can be simplified as follows:

(14) $$\begin{array}{l} u_{x}=\frac{P}{4\pi G}(\frac{xz}{\rho^{3}}-(1-2\nu )\frac{xz}{\rho (\rho +z)})\\ u_{y}=\frac{P}{4\pi G}(\frac{yz}{\rho^{3}}-(1-2\nu )\frac{z}{\rho (\rho +z)})\\ u_{z}=\frac{P}{4\pi G}(\frac{z^{2}}{\rho^{3}}-2\frac{1-v}{\rho }) \end{array}$$

where *P* is the normal force loaded on the surface, and *ρ* is the distance between the inspected point and the original point.

Equation (14) implies that with regard to the known normal force loaded on, the corresponding deformation can be found. Based on the superposition principle, the shear displacement and the normal component of a particular point are equal to the integration from the force loaded on this point.

In view of the principles above, the situation in which the force components along the *Z* direction (normal force *Fz*) and *X* direction (shear force *Fx*) are loaded on the flexible tactile sensor model is studied and discussed in this paper. In order to simplify the analysis, the tactile sensor is treated as an elastic half-space regardless of the boundary effect. Based on the above analysis, this paper concentrates on the high-dimensional information decoupling of the force-sensitive flexible tactile sensor and approximating the high dimensional non-linear mapping relationship between the resistance signal and external force by the improved BP algorithm with a strong non-linear approximation ability, so as to improve the decoupling accuracy and sensitivity of the tactile sensor.

### 2.6. The Improved BP Algorithm

The BP algorithm is one of the most famous algorithms in the machine learning field [24]. The learning process of the BP algorithm consists of information forward-propagation and error backward-propagation [25]. During the forward-propagation process, the input signal is propagated from the input layer to the hidden layer, and then arrives at the output layer. During the backward-propagation process, the output errors are propagated from the output layer to the hidden layer, and then arrive at the input layer. All of the connecting weights would be adjusted during this process. If the output error does not satisfy the target value, the BP algorithm continues to be iterated until the mean square error is less than the pre-set minimum value, which means that the algorithm converges. A three-layer BP network algorithm can accomplish any non-linear mapping from the n-dimensional input space to the m-dimensional output space [26].

In this paper, the gradient descent with the momentum factor method is used to improve the performance of the standard BP algorithm. The method utilizes the input vector, errors, learning rate, and momentum factor, to compute the changing weight values. The weights are modified as in Equation (15). Adding the momentum factor during the learning process can reduce the vibration and improve the convergence rate for the BP algorithm:

(15) $$v_{jt}(N+1)=v_{jt}(N)+\alpha [(1-\eta )g(N)+\eta g(N-1)]$$

where *g*(*N*) represents the negative gradient at time *N*; *α* is the learning rate; and *η* is the momentum factor, 0 < *η* < 1.

In our decoupling process for the tactile sensor model, the tan-sigmoid function and linear function are taken as the activation functions for the BP algorithm. The input of our BP algorithm model is an *n*-dimensional vector consisting of the resistances from the conductive pillars, and the output is an *m*-dimensional vector consisting of the force components loaded on the tactile sensor.

In the experiment, we also use the Levenberg–Marquardt (LM) method with a fast convergence rate and strong robustness to optimize and train the BP algorithm, which is also called the LMBP algorithm. During the decoupling process, the LMBP algorithm is conducted to approach the relationship between the resistances and force components *Fz* and *Fx*, respectively. In the following sections, the *Fz* and *Fx* loaded on the tactile sensor are decoupled with high precision in different ways. The improved BP algorithm is used as an approximate machine to approach the mapping relationship between the resistance value of the force-sensitive conductive pillar and the force loaded on the sensor.

## 3. Decoupling Results and Discussion

### 3.1. Simulation

**Figure 6:**
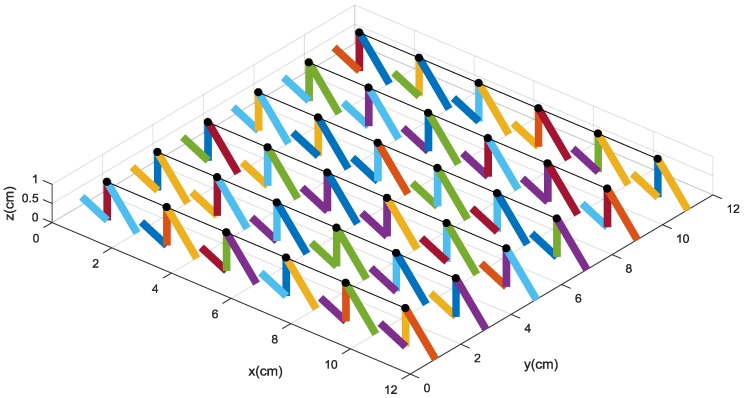
The model of a 6 × 6 sensor array.

In the numerical simulation, the 6 × 6 tactile sensor array is developed as shown in Figure 6. The black circles denote the original status of the upper sensing electrodes labeled “2”. When external force is loaded on the surface of the sensor, the resistances of the conductive pillars can be gained by scanning the circuit. Then, resistances are used to resolve the deformation and decouple the force components of the sensor by the intelligent decoupling algorithm. In order to emulate the real deformation of the tactile sensor and reduce the decoupling errors, we utilize the optimized BP algorithm with strong non-linear approximation ability to decouple the relationship between the resistance values and the force components along the *Z* direction (normal force *Fz*) and the *X* direction (shear force *Fx*), respectively.

**Figure 7:**
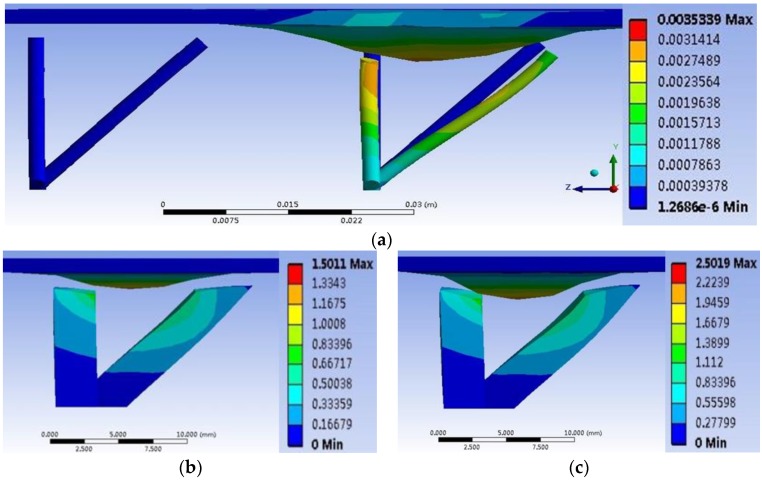
(**a**) The deformation under 1 MPa of pressure; (**b**) the deformation under 3 MPa of pressure; (**c**) the deformation under 5 MPa of pressure.

We also utilize the finite element analysis to analyze and simulate the performance of the tactile force-sensitive unit made by conductive rubber. During the simulation, 1 MPa, 3 MPa, and 5 MPa of pressure are loaded on the stress region, respectively, and the deformation of the sensitive unit is shown in Figure 7. The results imply that with the larger pressure, the larger and more obvious deformation of the soft sensitive unit is produced around the region where the press is loaded on. It also verifies Equation (14). Furthermore, according to the range of deformation generated by the stress, it is concluded that if the stress loaded on the area is around 1 cm^2^, the distance between the adjacent sensitive units should be around 1–2 cm; otherwise, it is very difficult for the sensitive units to feel or react to the touch force or pressure that is a little farther away from them, and then the deformation could not be detected.

### 3.2. Decoupling Process of Tactile Force Based on the Improved BP Algorithm with Different Hidden Nodes

In this paper, the BP algorithm model we constructed has three layers. First is the input layer, which consists of resistance values from the sensitive pillars. The resistance values are used to get the deformation and decouple the force loaded on the sensor array through the improved BP decoupling algorithm. Second is the hidden layer. In our experiments, the different hidden node numbers of the hidden layer are taken to verify the influence of the hidden node number works on the BP algorithm performance. The last one is the output layer, which consists of force vectors.

As to the shear force component along the *X*-direction (*Fx*) loaded on the surface of the sensor, the input vector of the BP algorithm is $Rx=[R_{x}^{1},R_{x}^{2},\ldots ,R_{x}^{36}]$, and the output vector is $Fx=[F_{x}^{1},F_{x}^{2},\ldots ,F_{x}^{36}]$, where *Fx* ∈ [0 N, 10 N]. Accordingly, as to the normal force component along the *Z* direction (*Fz*), the input vector is $Rz=[R_{z}^{1},R_{z}^{2},\ldots ,R_{z}^{36}]$, and the output vector is $Fz=[F_{z}^{1},F_{z}^{2},\ldots ,F_{z}^{36}]$, where *Fz* ∈ [0 N, 10 N]. $R_{z}^{k}$ and $R_{x}^{k}$ indicate the resistances of the conductive pillars labeled “1” and “4” from the *k*-th microstructure sensitive unit in the tactile sensor array. Namely, the *Rz* and *Rx* vectors include the 36 resistances coming from the 36 sensitive units, respectively. Meanwhile, $F_{z}^{k}$ and $F_{x}^{k}$ mean the force component applied on the sensitive pillar labeled “1” and “4” of the k-th sensitive unit, respectively. Therefore, the *Fz* and *Fx* vectors include the 36 force components along the *Z* direction and the *X* direction, respectively.

**Figure 8:**
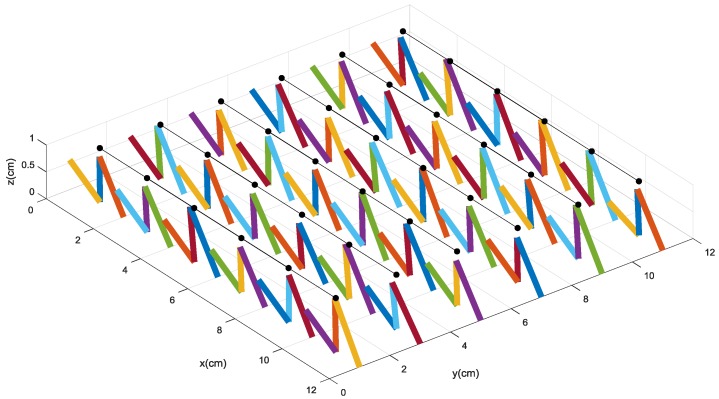
The deformation of the sensitive units when the force components are applied on it.

When the force components along the *X* direction and the *Z* direction are loaded on the sensor, the tactile-sensing array could be deformed. One of the deformation statuses of the tactile sensor array is shown in Figure 8, in which the original position of the upper sensing electrodes labeled “2” are represented by the black circles.

The BP algorithm model we constructed is improved by the LM method. The external force loaded on the flexible tactile sensor can be decoupled by the improved BP algorithm through the sensitive pillar resistances. During the numerical experiments, 2000 training samples and 200 testing samples are used to train and test the improved BP algorithm model, respectively. Each sample consists of a 36-dimensional resistance vector and a 36-dimensional force component vector. In the numerical experiments, the MATLAB 2016b neural network toolbox is used to establish the BP algorithm model.

The optimized number of hidden layer nodes plays a very important role in decoupling the mapping relationship between the resistance and force by the BP algorithm. More hidden nodes definitely lead to a longer training time, but the performance of the BP algorithm may not get better. The following formula is widely used to calculate the number of hidden nodes:

(16) $$h=\sqrt{i+o}+c$$

where *i* and *o* represent the input nodes number and output nodes number, respectively, *c* is a constant whose value is between 1 and 10, and *h* is the hidden nodes number.

The improved BP model is used to learn and approximate the relationship between the input resistance signal and the output force signal. Two-thousand samples are applied to train the improved BP algorithm model. It is expected to minimize the errors, gain the optimal model, and decouple the force components with high precision. During the training process, the mean square error is settled at 0.00001. The 200 testing samples are used to evaluate the decoupling performance of the BP algorithm.

**Figure 9:**
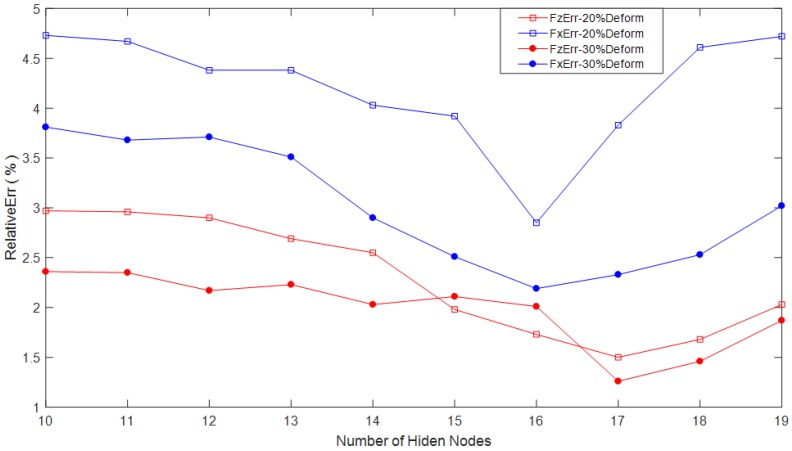
The decoupling results of *Fz* and *Fx* based on the back-propagation algorithm with different hidden nodes.

In the experiments, no more than 30% deformation of the sensitive pillars is conducted to the tactile sensor array. We use different hidden node numbers to verify the decoupling precision of the force components along the *X* direction and the *Z* direction, respectively. Under the premise that *i* = 36 and *o* = 36, in our experiments, the number of the hidden nodes is from 10 to 19. The average relative decoupling errors (*FxErr*, *FzErr*) of the force components *Fx* and *Fz* that are loaded on the tactile sensor based on the improved BP algorithm with different hidden node numbers are shown in Figure 9.

In Figure 9, the red squares and blue squares represent the relative decoupling errors of *Fz* and *Fx*, respectively, with no more than 20% deformation conducted to the sensitive pillars of the sensor array, separately. The red circles and blue circles denote the relative decoupling errors of *Fz* and *Fx*, respectively, with no more than 30% deformation conducted to the sensitive pillars, separately.

Fairly good decoupling results of the tactile force are obtained. The average relative decoupling errors of *Fz* and *Fx* with no more than 30% deformation of the conductive pillars based on 10 different hidden nodes are 1.98% and 3.02%, respectively. Correspondingly, the average relative decoupling errors of *Fz* and *Fx* with no more than 20% deformation are 2.30% and 4.21%, respectively. Thus, we concluded that all of the decoupling precisions for *Fz* are better than those for *Fx*. The main reason is that more exact information of deformation along the *Z* direction can be easily gained from the tactile sensor array by the sensitive pillars. The feature information of the deformation conducted by the shear force *Fx* is hard to be obtained and decoupled.

From Figure 9, it is also known that the relative decoupling errors for both *Fz* and *Fx* with 30% deformation are fairly better than those with 20% deformation. The more deformation conducted means the more force loaded, so, the more features of the force loaded on the sensor can be gained, which can improve the decoupling accuracy and reduce decoupling errors.

During the decoupling process, the decoupling errors are reduced by increasing the hidden node number firstly, but after the number increased to a certain value, even the hidden node numbers still keep increasing, and the decoupling errors would not be reduced again. For both *Fz* and *Fx*, the best decoupling results are gained when the hidden node numbers are 17 and 16. This verifies that the more hidden nodes, may not always gain the higher the accuracy.

### 3.3. Decoupling Process for Tactile Force Based on k-Cross Validation (k-CV) Method with Different Datasets

The *k*-CV method is widely used in machine learning algorithms to make the training set and testing set. For the *k*-CV method, the dataset should be divided into *k* subsets, and the experiment should be repeated *k* times. Each time, the *k*-1 subsets are used as the training set, and the remained subset should be used as the testing set. The final result of the *k*-CV method is the *k* times average result. Each subset should be acted as the testing set only once, and be used as the training set *k*-1 times.

We conduct the experiments with both large dataset samples and small dataset samples based on the BP algorithm with the 10-CV method, respectively. Experiments are repeated 10 times for both large samples and small samples. The large dataset consists of 2000 samples, and the small dataset consists of 200 samples.

As in Section 3.2, when the hidden node numbers are 16 and 17, the best decoupling result for *Fz* and *Fx* are gained, respectively. Therefore, when the 10-CV method is utilized to construct the dataset for a BP model, the hidden node numbers are set as 16 and 17 for decoupling *Fz* and *Fx* separately. Meanwhile, the experiment with less than 30% deformation and the length of the sensitive pillars is conducted.

**Figure 10:**
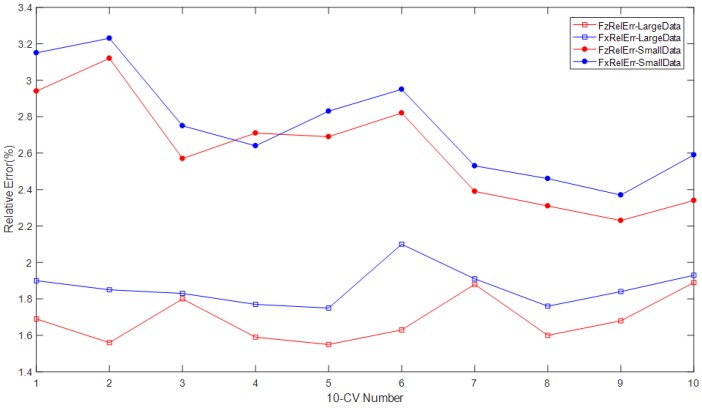
The relative decoupling errors of the 10-cross validation method with different datasets. During the decoupling process for *Fx* and *Fz*, the hidden node numbers are fixed at 16 and 17, respectively.

The decoupling results for both *Fx* and *Fz* are shown in Table 2 and Figure 10. They show the average relative decoupling errors of the force loaded on the tactile sensor array along the *X* direction (*Fx*) and the *Z* direction (*Fz*). Table 2 shows that each time, all of the decoupling results of the relative errors (both *Fx* and *Fz*) for the large dataset are much better than those for the small dataset. It means that even with the larger dataset, the better decoupling results can be gained by the suitable decoupling algorithm that we constructed. Although large datasets increase the complexity of the BP decoupling algorithm, it also includes more information and features about the resistances and forces; therefore, it can make the BP model more efficient:

(17) $$\begin{array}{l} FxRelErr=\sum_{t=1}^{36*Num}\left|\frac{F_{xt}^{\prime }-F_{xt}}{F_{xt}}\right|/(36*Num)\\ FzRelErr=\sum_{t=1}^{36*Num}\left|\frac{F_{zt}^{\prime }-F_{zt}}{F_{zt}}\right|/(36*Num) \end{array}$$

where $F_{zt}^{\prime }$ represents the decoupled result of force component *Fz* for the *t*-th testing sample, and *F_zt_* represents the actual force component loaded on the tactile sensor. *FzRelErr* and *FxRelErr* denote the average relative decoupling error of *Fz* and the *Fx*. *Num* is the number of testing samples, with each sample including the information of the 36 microstructure sensitive units.

The decoupling results shown in Figure 10 and Table 2 strongly imply that the relative decoupling errors based on the 10-CV method for force components *Fz* and *Fx* are much better than those that do not use the 10-CV method. The average relative decoupling errors for the large dataset are much better than those of the small dataset, as it contains more useful information regarding the features of the tactile sensor array. The decoupling results shown in Table 2 are much better than those presented in Section 3.2. In this section, the best average decoupling error for *Fz* is 1.69%, which is better than those that do not use the 10-CV method with the result of 1.98% presented in Section 3.2. The best average decoupling error for *Fx* in Table 2 and Figure 10 is 2.61%, which is also much better than those that do not use the 10-CV method with the decoupling result of 3.02% in Section 3.2.

**Figure 11:**
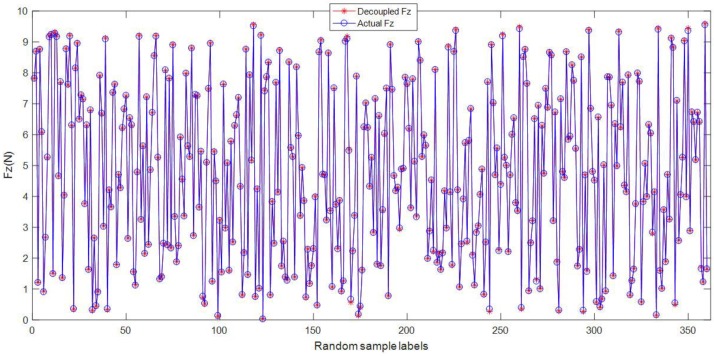
The decoupling results of *Fz* loaded on the tactile sensor by the improved BP algorithm.

In order to show the decoupling results further, 10 random samples are selected from the testing samples. Figure 11 shows the decoupling results of the 360 force components *Fz* from the random samples. The blue circles denote the actual force component applied on the sensor, and the red stars denote the decoupled force component by the improved BP algorithm. It is shown that the decoupled results of *Fz* are almost coincident with the actual force *Fz*, which implies that the decoupling accuracy based on the improved BP method is fairly good. The LMBP algorithm is very suitable to decouple the force applied on the tactile sensor. All of the decoupling results verify that the BP algorithm and the *k*-CV method play a very positive and effective part in the decoupling process for the tactile sensor array.

## 4. Conclusions

This paper presents an efficient intelligent BP decoupling algorithm for a novel flexible tactile sensor. Theoretical analysis and numerical experiments are conducted for the 6 × 6 flexible tactile sensor array. The mathematical model of the tactile sensor is constructed, and the relationship between the resistances and the force components is deduced. The LM method, momentum factor, learning rate, and different hidden nodes are used to improve the performance of the BP algorithm. The force components loaded on the tactile sensor are decoupled in high precision based on the improved BP algorithm. The *k*-CV method is used to optimize the performance of the datasets for the tactile sensor. The decoupling results also show that *k*-CV is a highly efficient method to improve the decoupling accuracy of the force components for the tactile sensor. A large dataset with the *k*-CV method is used to make the training and testing datasets gain a better decoupling result than the small dataset. All of the decoupling results indicate that the improved BP algorithm with high non-linear mapping ability has a fairly good performance in decoupling the force components for the tactile sensor array, and satisfies the requirement of the decoupling accuracy of the tactile sensor.

In future work, we will optimize and modify the decoupling method, and adjust the structure for the flexible tactile sensor. Then, to recognize the contact patterns that the three-dimensional force is loaded on, different scales and densities would be developed further to optimize the structure and performance of the tactile sensor array. The RBF algorithm could be used to improve the decoupling result of the three-dimensional force. In view of the strong non-linear mapping ability of the BP algorithm, the BP method will play an important role in the next works. As the MPa pressure can be conducted by atomic force microscope (AFM), the AFM tip technology would be used for the force control measurement and detection.

## Figures and Tables

**Table 1:** The parameters of the conductive rubber.

Young’s Modulus (MPa)	Poisson’s Ratio	Bulk Modulus (MPa)	Shear Modulus (MPa)
6.1	0.49	101.67	2.05

**Table 2:** The relative decoupling errors of the 10-cross validation (10-CV) method with different datasets.

10-CV	Relative Decoupling Errors of 10-CV Method			
	Large Dataset (2000 Samples)		Small Dataset (200 Samples)	
	*FzRelErr* (%)	*FxRelErr* (%)	*FzRelErr* (%)	*FxRelErr* (%)
1st	1.80	2.94	1.90	3.15
2nd	1.69	3.12	1.85	3.23
3rd	1.56	2.57	1.83	2.75
4th	1.59	2.71	1.77	2.64
5th	1.55	2.69	1.75	2.83
6th	1.63	2.82	2.10	2.95
7th	1.88	2.39	1.91	2.53
8th	1.60	2.31	1.77	2.46
9th	1.68	2.23	1.84	2.37
10th	1.89	2.34	1.93	2.59
Average Error (%)	1.69	2.61	1.86	2.75
